# Supplementary material for: Two pathways to resolve relational inconsistencies
Source: Sci Rep. 2025 Aug 21;15:30738. doi: 10.1038/s41598-025-16135-w (PMC12370936; doi:10.1038/s41598-025-16135-w)
Supplement: Supplementary file 1 — Supplementary Information. [file 41598_2025_16135_MOESM1_ESM.pdf]

Two pathways to resolve relational inconsistencies: Supplementary information

The results for other predictive features: Color and Number

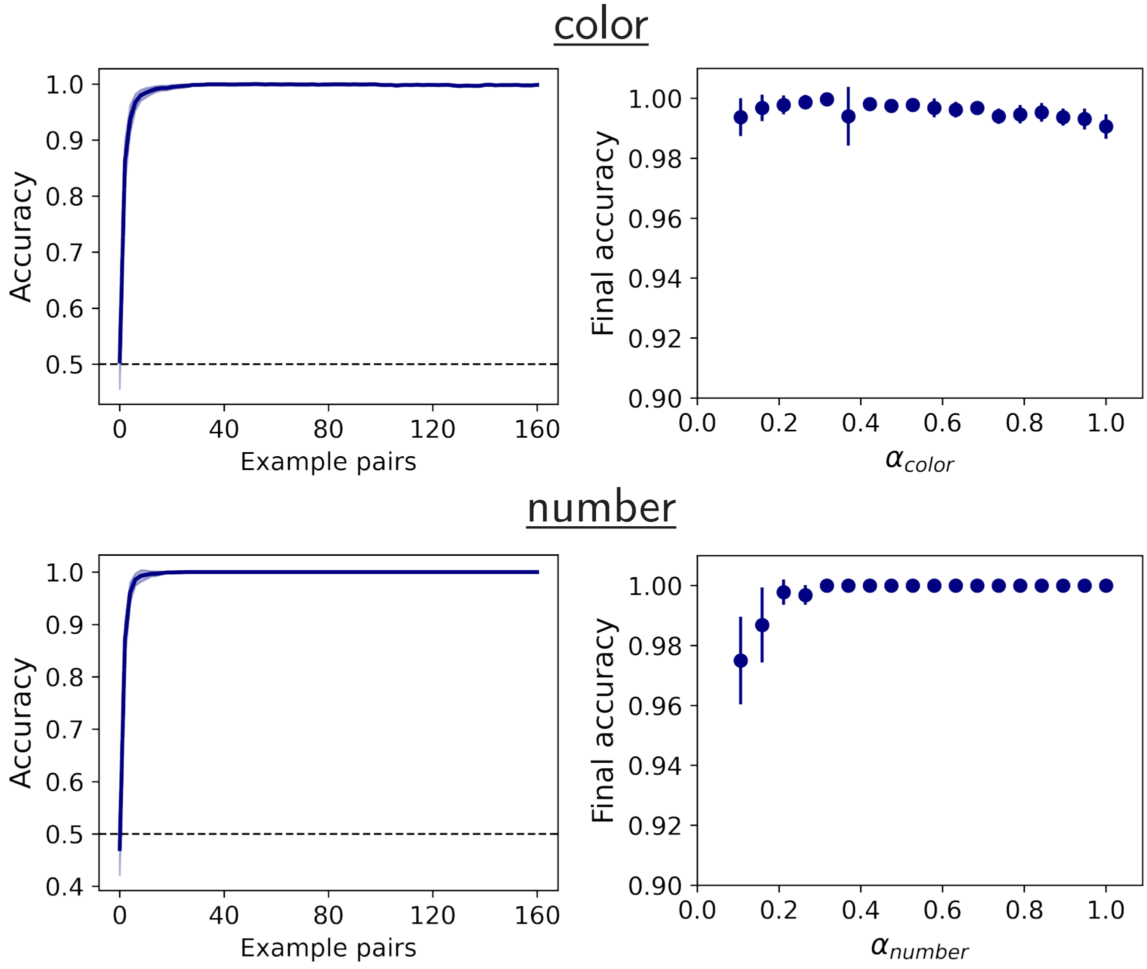

Figure S1: **Task performance.** Left: The average test accuracy of 100 networks trained on a task where the predictive features were color (top) or number (bottom). The change rule was $\alpha = 0.5$. Right: The final test accuracies for various values of α, averaged over 100 networks per α. Error shades and bars correspond to 95% CI.

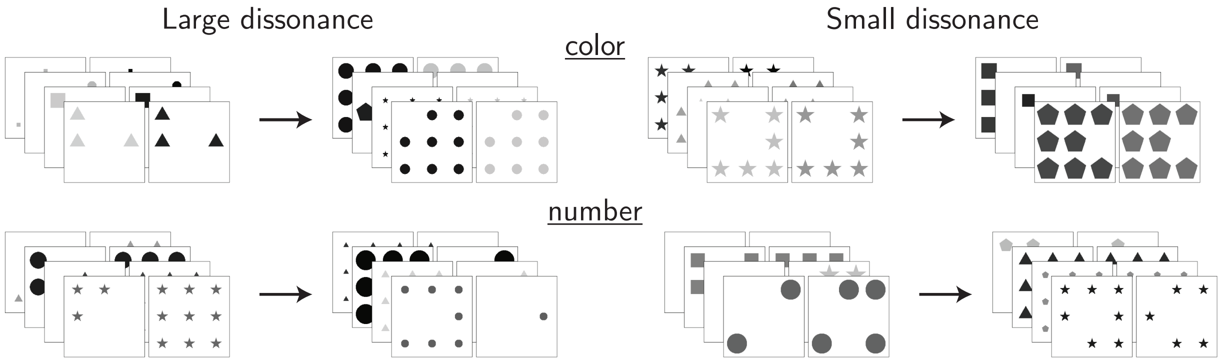

Figure S2: **Simulating a dissonance.** Initially, the predictive feature (top: color, bottom: number) increases by α. Then, the relationship is reversed to $-\alpha$. The dissonance magnitude is represented by α. Left: large dissonance $\alpha = 0.8$; Right: small dissonance $\alpha = 0.2$.

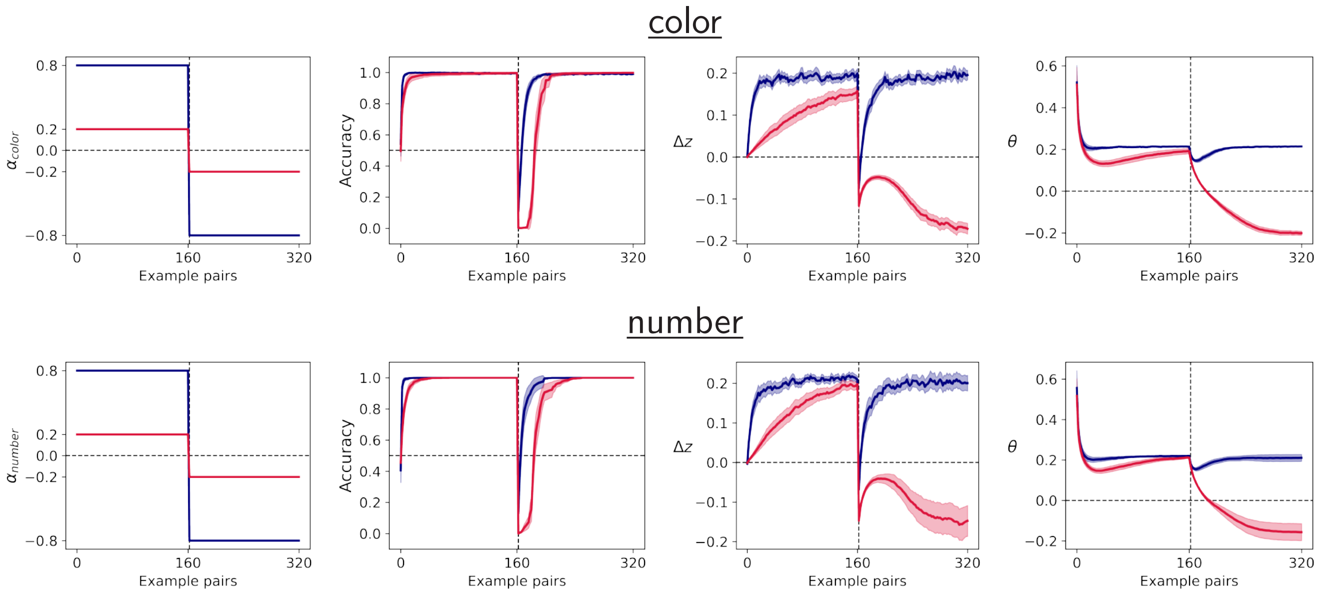

Figure S3: **Performance and adaptation following rule reversal.** 100 networks were presented with 160 examples in which $\alpha = 0.8$ (blue) or $\alpha = 0.2$ (red). Then, we reversed this rule, simulating a cognitive dissonance. From left to right, as a function of training examples: The rule α; Classification accuracy of the networks during the task; The ANNs' ΔZ; The ANNs' θ. The lines are the averages, and the shades correspond to 95% CI.

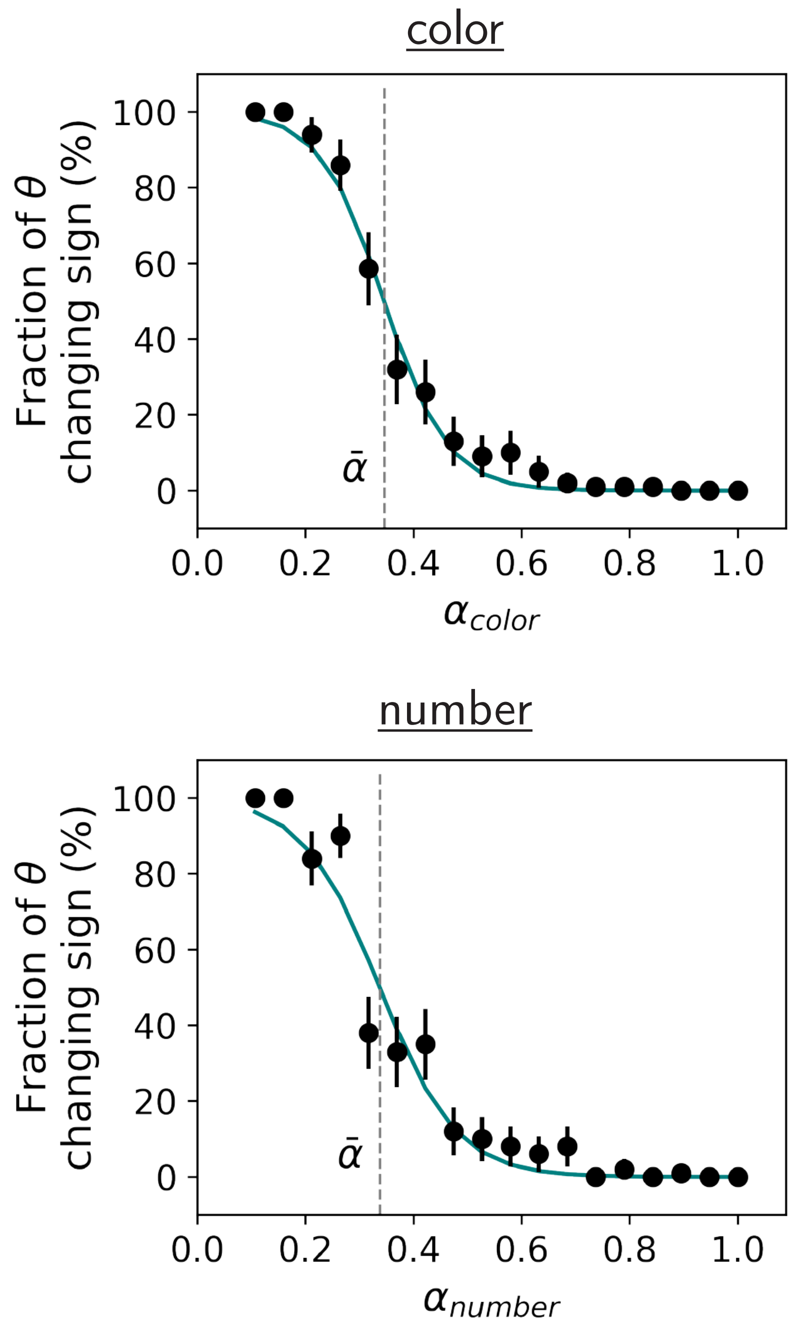

Figure S4: **Adaptation pathway versus dissonance magnitude.** For the color (left) and number (right) predictive feature, the percentage of networks that adapted their input representation ΔZ to match the expected θ increases a function of α. The inflection points between the adaptation pathways, $\bar{\alpha}$, were obtained by fitting the results (black) with a logistic function (green). Error bars correspond to 95% CI (Wilson estimation).

Fitting the adaptation pattern of the ANN

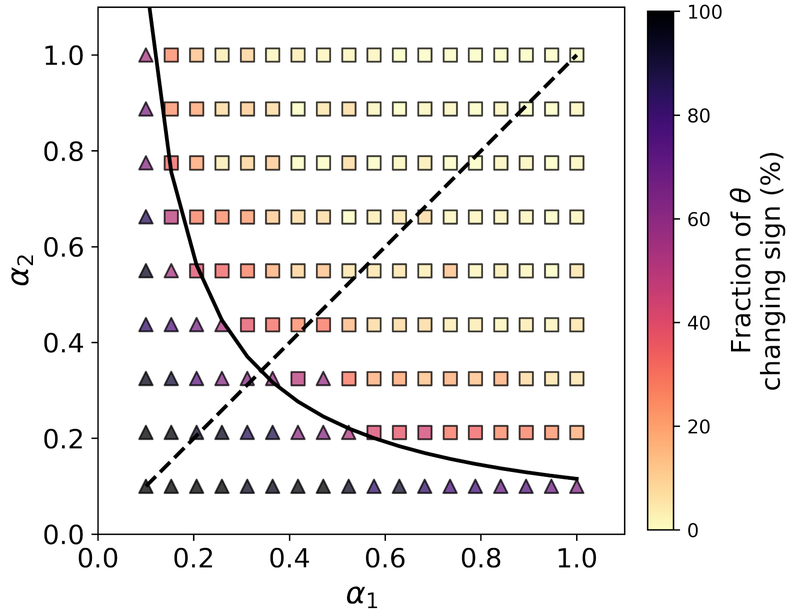

Figure S5: **Adaptation patterns for general rule reversals for the ANN.** The adaptation pattern, taken from (Fig. 5), with the predicted inflection line $\alpha_1\alpha_2 = \bar{\alpha}^2 = 0.34^2$ (black solid line). The value $\bar{\alpha}$ was obtained from the symmetric rule reversal case, represented by the dashed black line.
